# The Association of Healthy Eating Index and Sleep Disorders: A Meta‐Analysis

**DOI:** 10.1002/brb3.70640

**Published:** 2025-07-09

**Authors:** Wei Guo, Yibo Dong, Yang Xu, Yan Liu, Fengdan Wang, Sizhe Wang, Zibo Wu, Yuqi Gao, Bo Li

**Affiliations:** ^1^ Department of Epidemiology and Biostatistics, School of Public Health Jilin University Changchun P. R. China; ^2^ Jilin Provincial Center for Disease Control and Prevention (Jilin Academy of Preventive Medicine) Changchun China

**Keywords:** Healthy Eating Index (HEI), meta‐analysis, sleep quality, sleep time

## Abstract

**Background:**

Sleep disorders are problems that include both the quality and the time of sleep. It is related to overall diet quality, and dietary patterns also affect the quantity and quality of sleep in humans. The Healthy Eating Index (HEI) is widely used to assess the quality of diet. Epidemiological studies have investigated the association between diet quality and sleep, but these associations have not been confirmed in meta‐analyses. A meta‐analysis was performed to analyze whether there was an association between sleep disorders and HEI.

**Methods:**

A comprehensive search of several databases, including PubMed, Cochrane, Medline, Web of Science, and EMBASE, was conducted to identify relevant available studies reporting the relationship between sleep quality, abnormal sleep time, and HEI. Study results were meta‐analyzed using the odds ratios (ORs) and their 95% confidence intervals (95% CIs).

**Results:**

A total of five observational studies were involved in the final analysis. Meta‐analysis showed that higher HEI scores are associated with lower risk of poor sleep quality (OR = 0.860; 95% CI, 0.743–0.997; *p *= 0.045). The higher the HEI scores, the lower the risk of abnormal sleep time (OR = 0.296; 95% CI, 0.088–0.987; *p *= 0.048).

**Conclusion:**

The higher HEI score is associated with good sleep quality and a reduced risk of abnormal sleep time.

## Graphical abstract

Sleep disorders are associated with overall diet quality. The higher HEI scores are associated with good sleep quality and a reduced risk of abnormal sleep time.

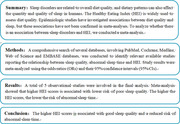

## 1 Introduction

Adequate sleep is essential for the regulation of the body's metabolism and various physiological functions. The National Sleep Foundation recommends that adults should get an appropriate amount of sleep, between 7 and 9 hours per day (Hirshkowitz et al. 2015). Sleep quality is about having enough energy to start the day and is influenced by a number of factors such as social, economic, lifestyle, and general health status (Hosseini et al. 2023). As a health epidemic issue, sleep disorders, such as sleep deprivation, insomnia, short or long sleep time, and poor sleep quality, have turned into health problems and have attracted the attention of world health agencies (Cheungpasitporn et al. 2017). The cumulative long‐term effects of sleep deprivation and sleep disorders are associated with harmful health consequences such as increased risk of hypertension, diabetes, obesity, and stroke (Cappuccio and Miller 2017). After decades of research, it can be demonstrated that sleep deprivation and sleep disorders have far‐reaching and widespread effects on human health (Institute of Medicine Committee on Sleep Medicine and Research 2006).

The assessment of diet quality is complex, and one widely used measure is the Healthy Eating Index (HEI), which is an indicator used to assess the conformity of a group of foods to the Dietary Guidelines of America (DGA) (Krebs‐Smith et al. 2018). Used as a tool for individual dietary allocation scores, it plays an important role in the association between diet and disease (González‐Treviño et al. 2022). HEI‐2010 is the most recent tool for assessing dietary quality as specified in the 2010 Dietary Guidelines for Americans and results in 12 components (Guenther et al. 2013). HEI‐2015 has the same components as HEI‐2010, except that empty calories are replaced with saturated fat and added sugars, resulting in 13 components (Krebs‐Smith et al. 2018).

Many studies have confirmed that sleep is linked to overall diet quality. Dietary patterns affect the time and quality of sleep in humans (Theorell‐Haglöw et al. 2020). For example, studies of Hispanic Americans (Mossavar‐Rahmani et al. 2017) and postmenopausal women (Stern et al. 2014) have shown that short sleep time is associated with lower HEI. A cross‐sectional study reported that insomnia severity and poor sleep quality were associated with lower diet quality and higher energy intake (Aslan Çin and Yardimci 2021). Kim et al. (2011) observed a U‐shaped association between sleep time and diet quality and eating behavior in the general adult population. However, one study found that short sleep time was not associated with dietary quality compared to women who had adequate sleep time (Xiao et al. 2016). The findings were inconsistent.

Despite growing evidence of an association between diet quality and sleep, no studies have so far assessed the relationship between sleep quality, sleep time, and HEI. In this study, we hypothesized that a higher HEI is associated with better sleep time/sleep quality. To resolve these disagreements, we conducted a meta‐analysis using HEI‐2010 and HEI‐2015 scores as measures to analyze whether there is an association between sleep disorders and HEI.

## 2 Materials and Methods

### 2.1 Registration

Our study protocol is registered with the International Prospective Register of Systematic Reviews (PROSPERO, https://www.crd.york.ac.uk/PROSPERO/); registration number: CRD42023401953.

### 2.2 Search Strategy

We systematically searched PubMed, Cochrane, Medline, Web of Science, and EMBASE databases (from database creation to January 2023). Boolean operators were used to combine the following MeSH terms and their entry terms, including sleep (and its variants), diet, and the HEI, to identify relevant available studies reporting the relationship between sleep disorders and HEI. The literature search was limited to reports in English and with human subjects by using the search terms(((((((((((((((((((((((((((sleep[MeSH Terms]) OR (sleep duration [Title/Abstract])) OR (sleep quality[Title/Abstract])) OR (sleep disorders[Title/Abstract])) OR (short sleep[Title/Abstract])) OR (hypersomnia[Title/Abstract])) OR (Short‐term sleep restriction[Title/Abstract])) OR (daytime sleepiness[Title/Abstract])) OR (long sleepers[Title/Abstract])) OR (short sleepers[Title/Abstract]))) OR (sleep initiation[Title/Abstract])) OR (maintenance disorders[Title/Abstract])) OR (habitual short sleepers[Title/Abstract])) OR (sleep deprivation[Title/Abstract])) OR (nap[Title/Abstract])) OR (napping[Title/Abstract])) OR (sleep disturbance[Title/Abstract])) OR (siesta[Title/Abstract])) OR (drowse[Title/Abstract])) OR (insomnia[Title/Abstract])) OR (drowsiness[Title/Abstract])) OR (24‐h sleep duration [Title/Abstract])) OR (night time sleep duration[Title/Abstract])) OR (short sleep duration[Title/Abstract])) OR (long sleep duration[Title/Abstract]))) AND (((((Diet, Healthy[MeSH Terms]) OR (HEI[Title/Abstract])) OR (HEI[Title/Abstract])). We also manually searched the reference lists of involved articles and previous reviews for potentially relevant studies. Study authors were not contacted by the researchers to identify additional studies.

Two researchers independently selected studies for full review based on the following inclusion criteria and extracted the final eligible literature: (1) the study population included all adults older than or equal to 18 years of age; (2) dietary patterns were defined using diet quality scores or indices or using statistical exploratory methods (e.g., pattern analysis); (3) sleep quality was assessed using the Pittsburgh Sleep Quality Index, Sleep Disorders Questionnaire, and self‐reported sleep time; (4)the relationship between poor sleep quality, abnormal sleep time, and HEI assessed; (5) odds ratios (ORs) or risk ratios (RRs) with 95% confidence intervals (CIs) or other data sufficient to calculate these numbers were provided; (6) Observational studies (cross‐sectional, case‐control, cohort).

### 2.3 Assessment of Dietary Quality

In this study, two scoring systems, HEI‐2010 and HEI‐2015, were applied and analyzed (Al‐Farhan et al. 2024). In the standardized score calculations, HEI‐2010 and HEI‐2015 effectively use the content per 1000 calories or percentage of total calories, as well as gender and age comparisons. High scores in the adequacy section indicate consistency with national recommendations, while the lowest scores above zero indicate that the nationally recommended adequacy score was not met. HEI “maximum” scores are assigned when moderate intake levels are at or above standard and when moderate intake levels are at or below standard. The average daily frequency of intake for each component of the HEI ranges from 0 to 10, and the total score ranges from 0 to 100 (Drenowatz et al. 2012; Feskanich et al. 2004; Kennedy et al. 1995). The HEI is categorized as “good” if it exceeds 80 points and is considered “poor” if the HEI is 50 points or less (Guo et al. 2004).

### 2.4 Data Extraction and Quality Assessment

Two researchers independently evaluated the studies and extracted the data, and any disagreements will be resolved through discussion. Data extraction for each article included first author, year of publication, country, sample size, diet assessment tool, sleep characteristic, key findings including ORs or RRs and 95% CIs, and quality scores for included studies.

### 2.5 Quality Assessment

The quality of the included literature was evaluated using the JBI scale, and the following information was assessed for each study: study purpose, study population, sample characteristics, statistical methods, and study value. A score of 0 was given for noncompliance; 1 for mentioning but not describing in detail; and 2 for a detailed and comprehensive correct description. We considered the literature to be of high quality when the quality score of the literature was greater than 12.

### 2.6 Statistical Analysis

Data from the study were collected to calculate OR and 95% CI, which will be used to measure the strength of the association between HEI and sleep disorders. Statistical heterogeneity was evaluated using Cochran's *Q* statistic and the *I* ^2^ statistic (Cochran 1954). If *p *> 0.05 for the *Q* test and *I* ^2^ < 50%, the heterogeneity was not significant, and the ORs and their 95% CIs of the outcome indicators were calculated using a fixed‐effects model (Cochran 1954). Otherwise, a random‐effects model was used. Methods of handling irregularities: the following measures were taken to address the issues identified in the analysis: (1) to explore the sources of heterogeneity, this study will perform a subgroup analysis of sleep time. (2) Publication bias was calculated using Egger's test, using the trim and fill procedure (sensitivity analysis) to rectify the results if there is publication bias. The methodology for identifying confounders in this study was based on the Cochrane Handbook for Systematic Reviews of Interventions (Higgins and Green 2010). All statistical analyses were performed using Stata version 14.0, and *p *< 0.05 was considered statistically significant.

## 3 Results

### 3.1 Literature Search and Study Characteristics

**FIGURE 1:**
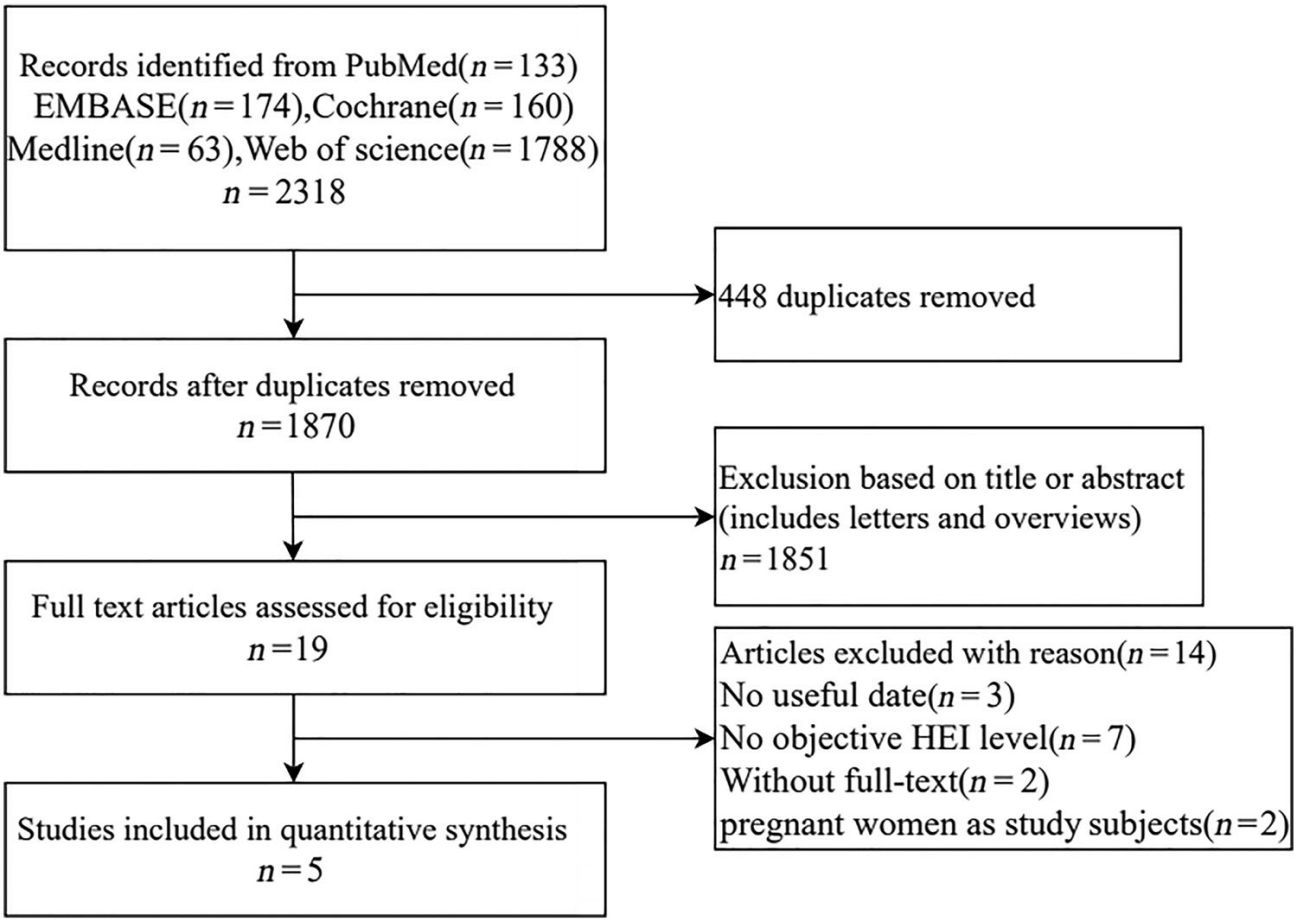
Flow diagram of the literature retrieval and selection for this study.

Figure 1 shows the flow diagram of the literature search and selection. The electronic search identified a total of 2318 studies, of which 133 were involved in PubMed, 160 in Cochrane, 174 in EMBASE, 63 in Medline, and 1788 in Web of Science. Among these studies, 1870 articles were retained after excluding duplicate literature, reviews, and letters. The remaining 23 studies were further screened by browsing through the title abstracts. During the literature screening process, we excluded letters and reviews at the title and abstract screening stage. These documents usually did not contain original research data and lacked a systematic description of the methodology and, therefore, did not meet the inclusion criteria. By reading through the full text, five studies (Behbahani et al. 2022; Daneshzad et al. 2022; Deng et al. 2022; Jansen et al. 2020; Xiao et al. 2016) met the inclusion criteria and were included in the analysis. To avoid missing any publications, all relevant studies were hand‐searched.

The basic characteristics and methodological quality of the included studies are listed in Table 1. All studies were observational; among the included studies, two studies occurred in Iran (Behbahani et al. 2022; Daneshzad et al. 2022), and two studies occurred in the United States (Deng et al. 2022; Jansen et al. 2020; Xiao et al. 2016). Two studies involved studies on sleep quality and the HEI (Daneshzad et al. 2022; Deng et al. 2022), two studies involved studies on sleep time and the HEI (Jansen et al. 2020; Xiao et al. 2016), and the other article deals with research on both sleep quality, and sleep time and the HEI (Behbahani et al. 2022). The methodological quality of all five papers was high. In subsequent analyses, we included sleep quality and sleep time data from the Behbahani study in the corresponding meta‐analyses so that a total of three studies were included in the sleep quality analysis and three studies in the sleep time analysis. To ensure that samples were not double‐counted, they were only counted once in the total sample size count. Total sample was not included in figures.

**TABLE 1:** Detailed information on included studies and main results: Association analysis based on adjusted odds ratio (OR) and relative risk (RR).

First author	Year	Country	Population	Sample size (*N*)	Diet assessment tool	Sleep characteristic	ORs/RRs (95% CI)	Quality score
Daneshzad	2022	Iran	Women	230	HEI‐2010	Sleep quality	0.080(0.032, 0.200)	16
Deng	2022	USA	Adult	22471	HEI‐2015	Sleep quality	0.913(0.912, 0.915)	14
Behbahani	2022	Iran	Adult	211	HEI‐2015	Sleep quality Sleep time	0.914(0.862, 0.969) 1.083(1.062, 1.104)	18
Jansen	2020	USA	Adult	12930	HEI‐2015	Short sleep time Long sleep time	0.190(0.064, 0.560) 0.310(0.113, 0.852)	16
Xiao	2017	USA	Women	896	HEI‐2010	Short sleep time Long sleep time	0.135(0.006, 3.320) 0.014(0.000, 0.607)	15

*Note*: The ORs and RRs reported in this table are multivariate adjusted to reflect independent associations between exposure factors and outcome indicators. All effect sizes are expressed as 95% confidence intervals, with intervals not containing 1 indicating statistical significance (*p* < 0.05).

### 3.2 Meta‐Analysis Result

We conducted a meta‐analysis of sleep disorders and the HEI to determine if there was an association between the two. We divided the sleep disorders into sleep quality and sleep time and analyzed them separately with the following findings.

#### 3.2.1 Sleep Quality and HEI

**FIGURE 2:**
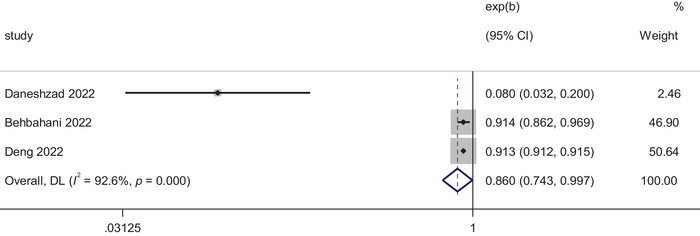
Forest plot of sleep quality and HEI.

According to Figure 2, based on the results of the heterogeneity test (*I* ^2^ = 96%, *p *< 0.01), there was a high degree of heterogeneity in the data, so we chose to use a random effects model as the primary analysis to report on the analysis of the association between HIE and poor sleep quality. The difference was statistically significant (OR = 0.860; 95% CI, 0.743–0.997; *p = *0.045). This indicated that higher HEI is associated with a lower risk of poor sleep quality.

#### 3.2.2 Sleep Time and HEI

**FIGURE 3:**
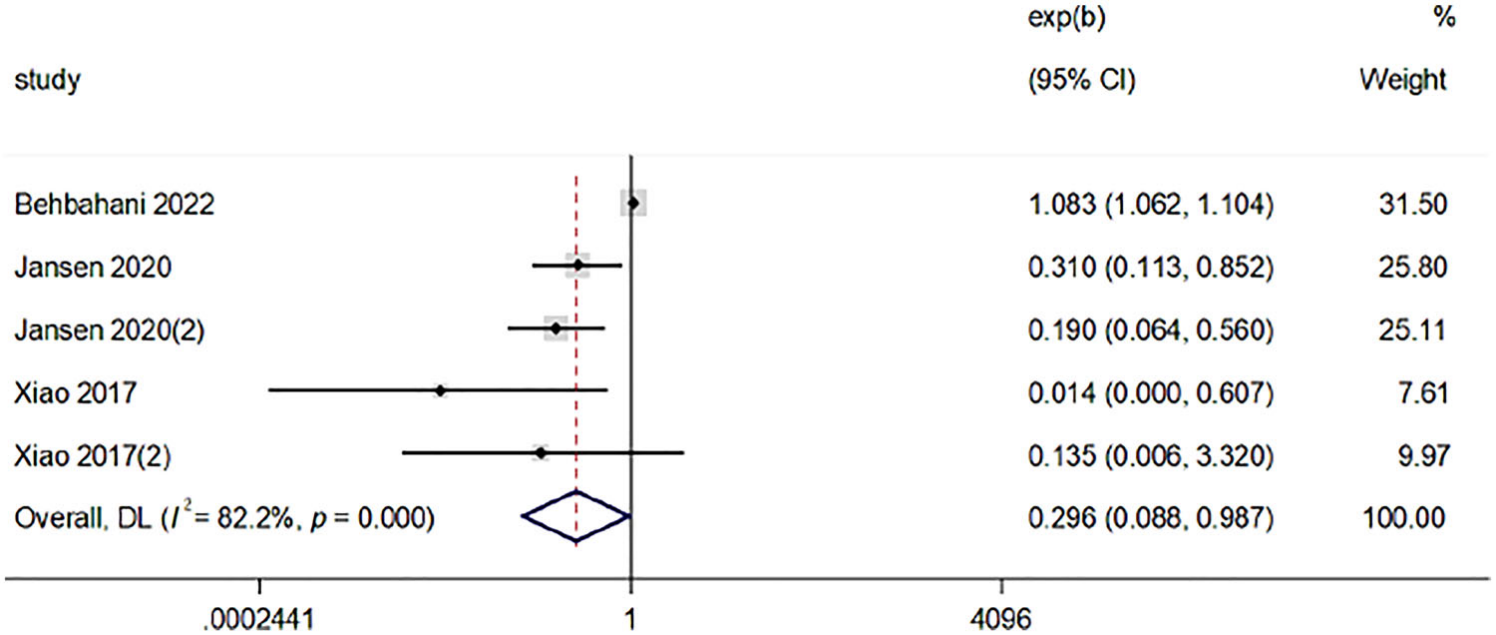
Forest plot of sleep time and HEI.

Figure 3 showed that estimates from the combined results of the four studies showed an association between HEI and abnormal sleep time using a random effects model with a heterogeneity test *I* ^2^ = 96%, *p *< 0.01. The difference was statistically significant (OR = 0.296; 95% CI, 0.088–0.987; *p = *0.048). This suggested that the higher the HEI, the lower the risk of abnormal sleep time. (In the figure, Xiao et al. 2016 and Jansen et al. 2020 represent short sleep time; Xiao et al. 2016 (2) and Jansen et al. 2020 (2) represent long sleep time.)

#### 3.2.3 Subgroup Analysis of Sleep Time

**FIGURE 4:**
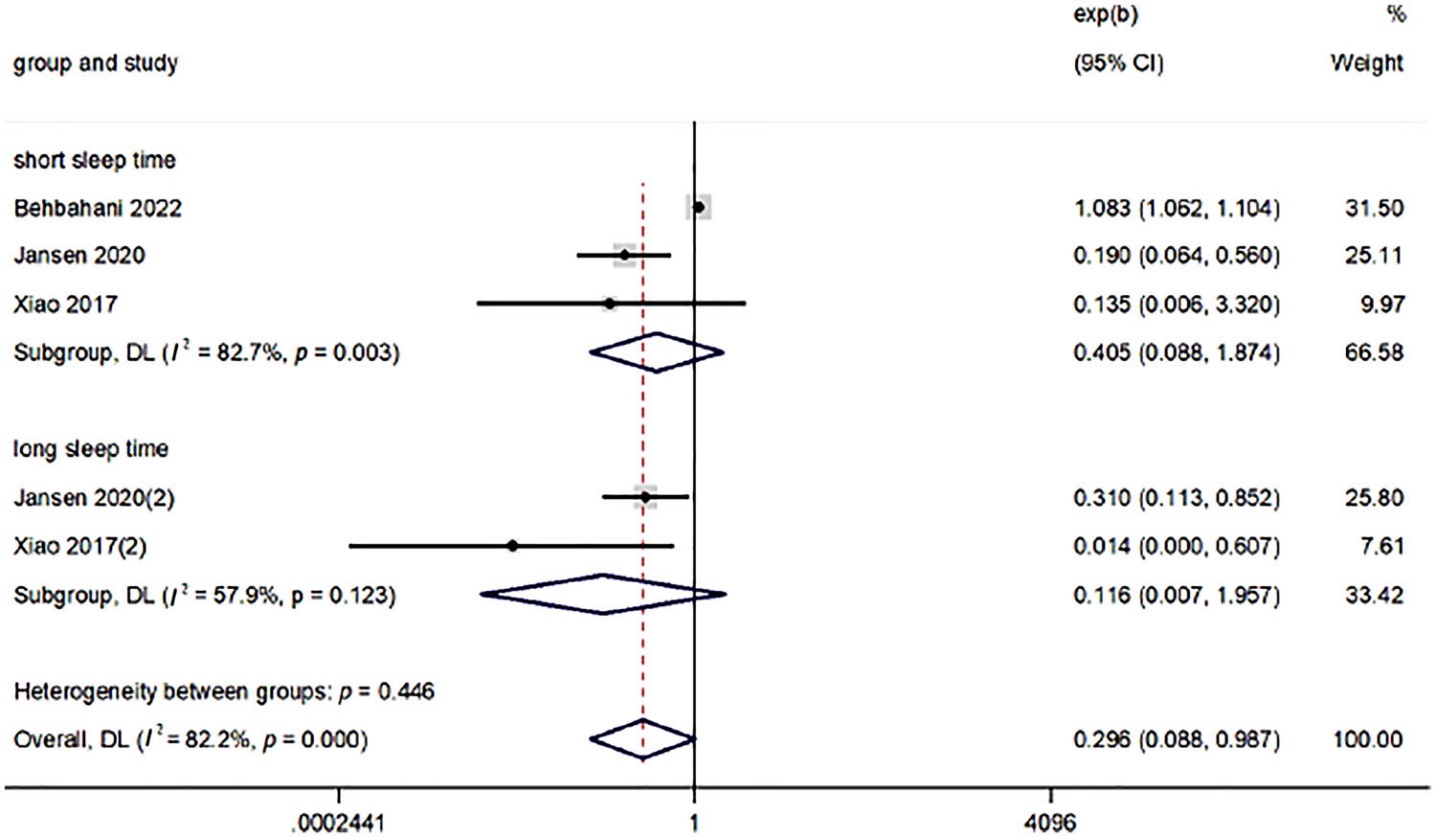
Comparison of forest plots with long sleep time and short sleep time.

Figure 4 showed that there was a high level of heterogeneity between studies, and a subgroup analysis was carried out to identify the main parameters involved in heterogeneity. Sleep time is divided into long sleep time and short sleep time, and the criteria for too long or too short sleep time are based on the criteria used to define sleep time in the included literature. When analyzed separately, the correlations between short/long sleep time and HEI were found to be non‐significant, with OR (95% CI) of 0.405 (0.088, 1.874) and 0.116 (0.007, 1.957), respectively.

### 3.3 The Publication Bias of the Included Studies

Publication bias was assessed using funnel plots and Egger's test, as shown in Table 2. Publication bias was observed in studies reporting sleep time. However, there was no significant difference in OR before and after trimming and filling, so we considered the risk of publication bias to be low and the results stable. Although the small number of studies may affect the robustness of the analysis, the methodology remains informative in the initial assessment of publication bias.

**TABLE 2:** Publication bias (Egger's test) and sensitivity analysis (trim and fill method) of studies.

	Egger test (*t, p*)	Number of trim and fill	OR (95%CI), *p* ^a^	OR (95%CI), *p* ^b^
Sleep quality	−1.01, 0.496	—	0.860 (0.743, 0.997), 0.045	—
Sleep time	−5.86, 0.010	0	0.296 (0.088, 0.987), 0.048	0.296 (0.088, 0.987), 0.048

^a^ Original variation.

^b^ Variation after trim and fill.

## 4 Discussion

This meta‐analysis discussed the association between HEI and sleep disorders, including sleep quality and sleep time. Overall, the results showed an association between HEI and sleep quality and abnormal sleep time, respectively. Our findings suggested that there is an association between higher HEI and good sleep. In the subgroup analysis, sleep time was divided into insufficient sleep time and long sleep time, and the results showed that none of the correlations between long/short sleep time and the HEI were significant.

Although it has not been elucidated how sleep disorders (including sleep quality and sleep time) interact with HEI, several possible mechanisms have been proposed. Circadian rhythms drive changes in metabolic patterns, and changes in metabolic and nutritional status can affect circadian rhythms (Vernia et al. 2021). A good circadian rhythm controls hormones. Related hormones involved in nutrient metabolism, such as glucagon, insulin, growth hormone‐releasing peptides, leptin, and corticosterone, are all associated with good circadian rhythms (Fonken and Nelson 2014). Nutrients in food may influence the production of hormones. For example, dietary factors may affect levels of lipocalin, a protein hormone involved in regulating glucose concentration and fatty acid catabolism, which affects circadian rhythms (Cornelissen 2018). Unhealthy dietary patterns may affect overall sleep quality by affecting the intake or consumption of certain nutrients (Zuraikat et al. 2020). Sleep not only affects diet and eating patterns but also feeds back to influence the sleep–wake regulation process itself (Laposky et al. 2008). Sleep deprivation alters the physiological regulation of metabolic hormones, and the daily cycle of sleep and wakefulness is regulated by various hormones produced by the hypothalamus and external stimuli (España and Scammell 2011; Jones 2011). Poor sleep is associated with changes in circulating concentrations of melatonin, cortisol, growth hormone‐releasing peptide, and leptin (Copinschi et al. 2014; Gonnissen et al. 2013; Knutson and Van Cauter 2008; Lin et al. 2020; Ulhôa et al. 2015).

We found that higher HEI is associated with a lower risk of poor sleep quality, and the current study found that in a study of US adults, diet quality and diet composition using the HEI‐2015 as a measure were found to be associated with sleep disturbances. Higher HEI scores, or better diet quality, may be associated with a reduced risk of sleep disorders (Deng et al. 2022). In addition, primary insomnia was found to be less likely to occur in people who adhered to a healthy eating pattern in some studies (Sadat et al. 2021). This is consistent with the findings of our study. We found that this may be due to the fact that components of a healthy diet are more helpful in initiating and maintaining sleep. For example, polyphenols in fruits and vegetables may affect sleep through the melatonin pathway (Noorwali et al. 2019) (melatonin is a natural antioxidant that is used to regulate circadian rhythms and sleep disorders) (Cajochen et al. 2003). Melatonin secretion plays a crucial role in the biological clock and circadian sleep–wake rhythms, as well as in the onset of sleep at night (Ongan and Yuksel 2017). Similarly, dairy products contain high levels of tryptophan, a dietary amino acid that is a precursor to brain serotonin, a sleep‐inducing agent (Yasuda et al. 2019). A prospective cohort study found that poor sleep quality was associated with greater food intake and a low‐quality diet. The possible reasons for this may be that poor sleep quality leads to excessive food and energy intake through stimulation of hunger and/or suppression of satiety signals (St‐Onge 2017). Furthermore, one mechanism by which sleep deprivation may increase food intake is by altering key appetite hormones (e.g., leptin, growth hormone‐releasing peptide, and cortisol) to enhance hunger (homeostatic feeding drive) (Bosy‐Westphal et al. 2008; Nedeltcheva et al. 2009; Omisade et al. 2010; Pejovic et al. 2010; Schmid et al. 2009; Simpson et al. 2010). Sleep deprivation is associated with increased levels of growth hormone‐releasing peptide, decreased levels of leptin, altered expression of many hypothalamic neuropeptides such as neuropeptide Y (NPY) and pro‐adrenocorticotropin (POMC), and increased food intake in humans (Fujihara et al. 2003; Koban et al. 2008; Spiegel et al. 2004).

Furthermore, the higher the HEI, the lower the risk of abnormal sleep time. Our analysis of possible mechanisms was due to sleep also influencing choices about diet. People who had poor sleep quality and abnormal sleep time were more likely to choose an unhealthy diet. People who slept less preferred energy‐rich foods (such as fats and refined carbohydrates) (Beebe et al. 2013; Kjeldsen et al. 2014; Peuhkuri et al. 2012; Smith et al. 2016), ate fewer vegetables, and ate at unconventional times. People with long sleep times and short sleep times generally ate irregular meal times, and snacking dominated their meals.

However, the association between short and long sleep time and HEI was found to be insignificant. As was a subgroup analysis in the USA using nationally representative data on women up to 5 years after delivery, which concluded that there was no association between short sleep time and diet quality (Xiao et al. 2016). This is inconsistent with previous studies (Mossavar‐Rahmani et al. 2017; Stern et al. 2014). The possible reason for our analysis was that the number of included studies was too small and heterogeneous to provide a meaningful pooled analysis.

The current meta‐analysis had the following advantages. First, to our knowledge, this was the first meta‐analysis on the relationship between HEI and sleep disorders (including sleep quality and sleep time). Second, we chose two different outcomes for the analysis, poor sleep quality and sleep time, and both concluded that there was an association with the HEI. However, several restrictions apply to this meta‐analysis. First, a major limitation of this meta‐analysis is the small number of included studies, which limits the robustness of the subgroup analyses, and results need to be interpreted with caution. Despite our best efforts to search multiple databases, research in this area is still at an early stage. Future studies should increase the sample size to improve the reliability of the analysis. We plan to update the analysis as more data becomes available. Higher heterogeneity (e.g., higher *I* ^2^ values) was observed in some of the subgroup analyses, which may stem from differences in study design, population characteristics, or interventions. We have reduced the effect of heterogeneity through sensitivity analyses and random effects models. Secondly, the included studies were cross‐sectional, allowing for the establishment of associations between variables but not for determining the causality or directionality of the relationship between sleep disturbance and HEI. Therefore, further high‐quality cohort studies and randomized controlled trials (RCTs) were needed to validate this relationship.

## 5 Conclusion

In conclusion, this meta‐analysis showed that there was a significant correlation between HEI and sleep disorders (including sleep quality and sleep time). There is an association between higher HEI and good sleep. New evidence is provided for the study of sleep and diet. It is recommended that more high‐quality and well‐designed longitudinal studies and field trials should be conducted in the future to confirm these findings.

## Author Contributions

**Wei Guo**: methodology, writing ‐ review and editing, funding acquisition. **Yibo Dong**: conceptualization, writing – original draft, writing – review and editing. **Yang Xu**: software, writing – review and editing, methodology. **Yan Liu**: conceptualization, visualization, writing – review and editing. **Fengdan Wang**: investigation, methodology. **Sizhe Wang**: investigation, writing – review and editing. **Zibo Wu**: methodology, writing – review and editing. **Yuqi Gao**: writing – review and editing, data curation. **Bo Li**: writing – review and editing, supervision, resources.

## Ethics Statement

The authors have nothing to report.

## Conflicts of Interest

The authors declare no conflicts of interest.

## Peer Review

The peer review history for this article is available at https://publons.com/publon/10.1002/brb3.70640

## Data Availability

All data generated or analyzed during this study are included in this published article [and its supplementary information files].
